# Risk of Enteric Infection in Patients with Gastric Acid Supressive Drugs: A Population-Based Case-Control Study

**DOI:** 10.3390/jpm11111063

**Published:** 2021-10-22

**Authors:** Chia-Jung Kuo, Cheng-Yu Lin, Chun-Wei Chen, Chiu-Yi Hsu, Sen-Yung Hsieh, Cheng-Tang Chiu, Wey-Ran Lin

**Affiliations:** 1 Department of Gastroenterology and Hepatology, Linkou Chang Gung Memorial Hospital, Taoyuan 333, Taiwan; m7011@cgmh.org.tw (C.-J.K.); 8805035@cgmh.org.tw (C.-Y.L.); 8902088@cgmh.org.tw (C.-W.C.); siming.shia@gmail.com (S.-Y.H.); ctchiu@adm.cgmh.org.tw (C.-T.C.); 2 Department of Gastroenterology, Chang Gung Memorial Hospital at Linkou, College of Medicine, Chang Gung University, Taoyuan 333, Taiwan; 3 Chang Gung Microbiota Therapy Center, Linkou Chang Gung Memorial Hospital, Taoyuan 333, Taiwan; 4 Center for Big Data Analytics and Statistics, Linkou Chang Gung Memorial Hospital, Taoyuan 333, Taiwan; joy960111@gmail.com; 5 Department of Gastroenterology and Hepatology, Chang Gung Memorial Hospital, 5, Fushin Street, Kweishan, Taoyuan 333, Taiwan

**Keywords:** enteric infection, proton pump inhibitor, H2-receptor antagonists

## Abstract

Long-term use of gastric-acid-suppressive drugs is known to be associated with several adverse effects. However, the association between enteric infection and acid suppression therapy is still uncertain. This study aimed to evaluate the association between gastric acid suppression and the risk of enteric infection. **Materials and Methods:** We conducted a population-based case-control study using the data from Chang Gung Research Database (CGRD) in Taiwan. Between January 2008 and December 2017, a total of 154,590 adult inpatients (age > 18) were identified. A pool of potential eligible controls according to four propensity scores matching by sex, age, and index year were extracted (*n* = 89,925). Subjects with missing data or who received less than 7 days of proton pump inhibitors (PPIs) and/or H2-receptor antagonists (H2RAs) were excluded. Finally, 17,186 cases and 69,708 corresponding controls were selected for analysis. The use of PPIs and H2RAs, the result of microbiological samples, and co-morbidity conditions have been analyzed. Confounders were controlled by conditional logistic regression. **Results:** 32.84% of patients in the case group used PPIs, compared with 7.48% in the control group. Of patients in the case group, 9.9% used H2RAs, compared with 6.9% in the control group. Of patients in the case group, 8.3% used a combination of PPIs and H2RAs, compared with 2.7% in the control group. The most common etiological pathogens were *Enterococcus* (44.8%), *Clostridioides difficile* (34.5%), and *Salmonella* spp. (10.2%). The adjusted odds ratio (OR) for PPI use with enteric infection was 5.526 (95% confidence interval [CI], 5.274–5.791). For H2RAs, the adjusted odds ratio was 1.339 (95% confidence interval [CI], 1.261–1.424). Compared to the control group, persons with enteric infection had more frequent acid-suppressive agent usage. **Conclusions:** This study demonstrates that gastric-acid-suppressive drug use is associated with an increased risk of enteric infection after adjusting for potential biases and confounders.

## 1. Introduction

Proton pump inhibitors (PPIs) inhibit the secretion of hydrogen ions into the stomach by inhibiting the H+/K+ ATPase enzyme present in gastric parietal cells. The action results in prolonged elevation of intragastric pH levels and is commonly used to treat gastric-acid-related diseases such as upper gastrointestinal bleeding, peptic ulcers, and gastroesophageal reflux [1,2,3]. PPIs are considered well tolerated and highly efficacious. However, emerging studies have suggested that long-term use of PPIs may be associated with several adverse effects, such as bacterial pneumonia, osteoporotic-related fractures, kidney disease, impaired absorption of nutrients, ischemic stroke, cardiovascular events, and even, the risk of cancer [1,4,5,6,7,8,9,10,11,12]. Long-term acid suppression facilitates the development of fundic ECL cell hyperplasia, overgrowth of non-Helicobacter pylori bacterial species, lower the acid bactericidal effects for harmful microorganisms and alters the natural course of Helicobacter pylori gastritis, transforming the antral-predominant pattern into a body-predominant pattern [13]. A link between gastric-acid-suppressive drugs and increased enteric infection risk is based on several potential hypotheses. By reducing the secretion of hydrochloric acid produced by the stomach, PPIs and H2RAs may promote the growth of gastrointestinal pathogenic microflora, increase bacterial translocation, affect the gastrointestinal microbiome, PPIs therapy also inhibited the neutrophil’s bactericidal activity [14]. 

Previous investigation in Western countries reported that there is an association between acid suppression drug use and increased risk of enteric infection [15]. Furthermore, the effect was related to the degree of gastric acid inhibition and greater for PPI use compared with H2RA use. There was a trend for the association to be stronger for *Salmonella*, *Campylobacter*, or *Shigella* infection [15]. The association between long -term PPIs use and *Clostridioides difficile* infection is also reported [16,17,18].

Metabolism of PPI depends on hepatic cytochrome P450 enzymes, especially the CYP2C19 genotype, and has different activity due to gene polymorphism. Genetic polymorphism of CYP2C19 shows marked interracial differences, with the poor metabolizer phenotype representing 2 ~ 5% of Caucasian and up to 11 ~ 23% of Oriental population [19]. Poor metabolism of PPI results in greater bioavailability and subsequently increased antisecretory efficacy.

Our current study aims to evaluate the association between gastric-acid-suppressive drug use and the risk of enteric infection for Asian population.

## 2. Materials and Methods

### 2.1. Ethics Statement

Ethics approval was obtained from the Institutional Review Board of CGMH (No.201900774B0C601). As all data were anonymized from existing databases and results were presented in aggregate, the requirement for informed consent was waived according to IRB regulations.

### 2.2. Data Source

Data were obtained from the Chang Gung Research Database (CGRD). The Chang Gung Medical Foundation (CGMF), which consists of seven Chang Gung Memorial Hospitals (CGMHs), is the largest medical system in Taiwan. CGMF has 10,070 beds and admits more than 280,000 patients per year. All seven CGMHs use electronic medical records (EMRs). The CGRD is an anonymized database comprising of multi-institutional standardized EMRsn [20]. This study is based in part on data from the CGRD provided by CGMHs. The interpretation and conclusions contained herein do not represent the position of CGMHs.

### 2.3. Study Population

**Figure 1:**
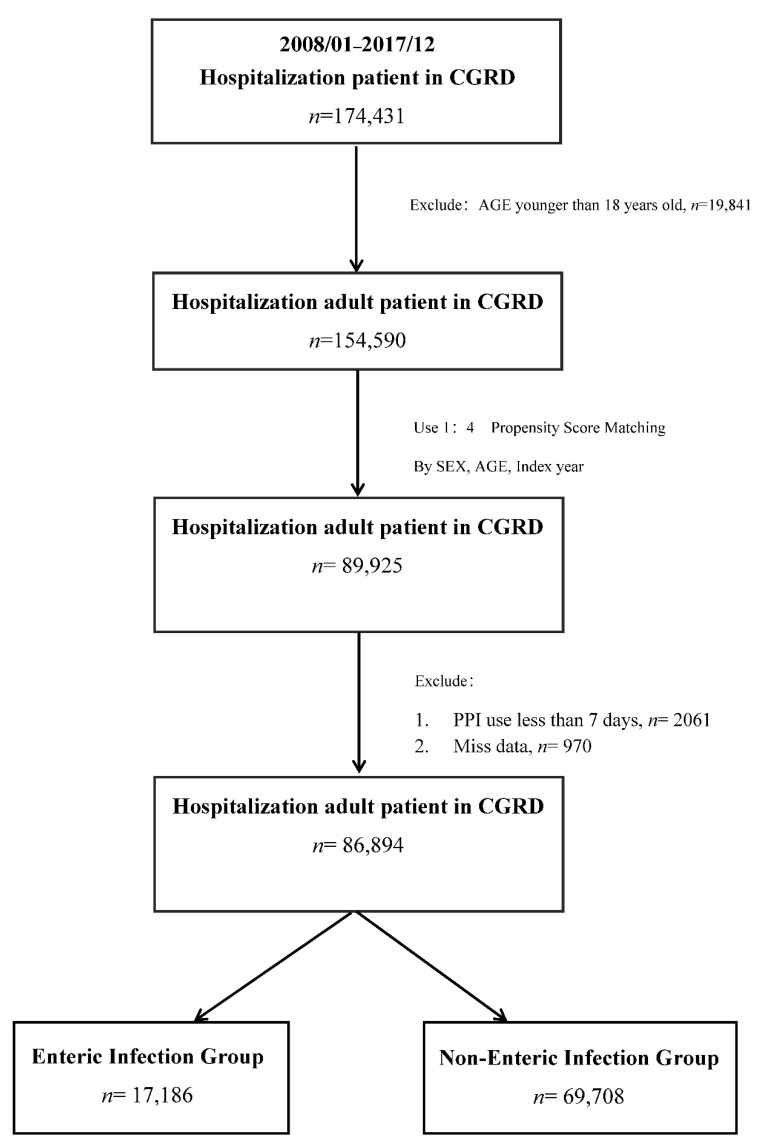
Flow chart of current study.

We conducted a case control study using data from CGRD in Taiwan. We used the International Classification of Diseases (ICD-9-CM and ICD-10-CM) to define diseases. Case subjects were identified from the CGRD by using inpatient discharge records. Between January 2008 and December 2017, a total of 154,590 adult inpatients (age > 18) were identified. A pool of potential eligible controls with the same follow-up period as the case patient according to 4 propensity scores matching by sex, age, and index year were extracted (*n* = 89,925). Because the therapeutic doses of PPIs or H2Bs reach a steady state after daily dosing and thus achieve their maximal effective level between 5 to 7 d, subjects who received less than 7 days of drugs were excluded. Finally, 17,186 cases and 69,708 corresponding controls were selected for analysis. The flow chart illustrates inclusion and exclusion in the current study (Figure 1).

### 2.4. Exposure Assessment

Information on the prescribed drugs was extracted from the CGRD. We identified all PPIs and H2RAs prescribed within 6 months before the index date; PPIs included pantoprazole, lansoprazole, rabeprazole, esomeprazole, and dexlansoprazole. H2RAs included famotidine and cimetidine. The defined daily doses (DDDs) recommended by the World Health Organization were used to quantify the use of PPIs or H2RAs. Cumulative DDD was estimated as the sum of dispensed DDD for any PPI or H2RA, and the final dose was defined as the latest dose taken within the specified period prior to the index date. Collected data included date of prescription, daily dosage, and number of days on the drug.

### 2.5. Statistical Analysis

For comparison, the chi-square statistics test was used. Crude and adjusted ORs with 95% confidence intervals (CIs) of exposure for enteric infection cases compared with control cases were estimated using conditional logistic regression. All analyses were performed using SAS statistical software (version 9.4 for Windows; SAS Institute, Inc., Cary, NC, USA).

## 3. Results

In total, we identified 17,186 cases of admitted patients with enteric infection and 69,708 applicable control cases. Case demographics are described in Table 1. Within the enteric infection group (case group), gender was represented as 51.9% male to 48.1% female. Over 50% of patients in the case group were older than 50 years of age. Of patients in the case group, 32.84% were on PPIs, compared to 7.48% in the control group; 9.9% of patient in the case group were on H2RAs, compared to 6.9% in the control group. Of patient in the case group, 8.3% were on combined PPIs + H2RAs therapy, compared to 2.7% in the control group. The most common comorbidities along with the case group were hypertension (54.9%) and diabetes mellitus (37.0%). The in-hospital all-cause mortality within the case group was 14.1%. 

Table 2 shows the association between exposure to PPIs or H2RAs and risk of enteric infection. The adjusted OR for PPIs use with enteric infection was 5.526 (95% CI, 5.274–5.791). For H2RAs, the adjusted odds ratio was 1.339 (95% CI, 1.261–1.424). A significant dose response was observed in PPIs or H2RAs use and risk of enteric infection. Subgroups with an OR above average were esomeprazole (OR, 6.319; 95% CI, 5.959 to 6.7) and pantoprazole (OR, 5.799; 95% CI, 5.297 to 6.349).

Table 3 shows the stratum-specific odds ratios (ORs) for the association between use of PPIs therapy and enteric infection for various subgroups of patients. Subgroups with an OR above average were female (OR, 5.565; 95% CI, 5.193–5.964), age between 18–65 year-old (OR 6.252–8.565) and patients with a diagnosis of diabetes mellitus (OR, 5.724, 95% CI, 5.328–6.15). 

Table 4 shows the stratum-specific ORs for the association between use of H2RAs therapy and enteric infection for various subgroups of patients. For subgroup of ischemic heart disease, acute myocardial infarction, liver cirrhosis/hepatitis, gastroesophageal reflux, gastric ulcer, duodenal ulcer and irritable bowel syndrome, there is no obvious association between use of H2RAs therapy and intestinal infection ( OR less than 1)

The most common etiological pathogens were *Enterococcus* (44.8%), *Clostridioides difficile* (34.5%), and *Salmonella* spp. (10.2%), as shown in Table 5.

## 4. Discussions

To our knowledge, this is the first large-scale cohort study investigating the association between gastric-acid-suppressive drug use and the occurrence of enteric infection for Asia population. A significant dose response was also observed The current study suggests that use of gastric-acid-suppressive drugs increases the risk of enteric infection. The adjusted odds ratio (OR) between PPI use and enteric infection was 5.526 (95% confidence interval [CI], 5.274–5.791). For H2RAs, the adjusted odds ratio was 1.339 (95% confidence interval [CI], 1.261–1.424).

Gastric acid is bactericidal and is an important defense mechanism against ingested microorganisms. PPIs, which decrease the secretion of hydrochloric acid, may inadvertently make the stomach a more hospitable environment to ingested pathogens. Gastric acid has a pH < 4 and is known to eliminate exogenous acid sensitive bacteria within 15 min. A recent study by sequenced 16S rRNA from a fecal sample revealed that oral microbiome is more abundant in the gut microbiome among those taking PPIs. Moreover, PPI-induced hypochlorhydria facilitates colonization of more distal parts of the digestive tract by upper gastrointestinal microbiota PPI use was associated with increases in the Lactobacillales order, and in particular the family Streptococcaceae [21,22,23].

A link between PPIs and increased enteric infection risk is based on several potential mechanisms. PPIs can alter the gut microbiota, leading to dysbiosis and impaired gut barrier function that results in compromise to gut immunity and susceptibility to various enteric pathogens [21,24]. PPIs negatively influence the function of polymorphonuclear cells, specifically with respect to phagocytosis, oxidative burst, chemotaxis, and cytotoxic activity, weakening the immune system. Gastric acid acts as a barrier to progression down the GI tract for pharyngeal and environmental bacteria. The usage of PPIs also affects the gut microenvironment by modifying pH in the stomach and small intestine, allowing colonisation by these bacteria further along the GI tract and is proven to cause gut dysbiosis. Moreover, PPIs are associated with a significant decrease in Shannon’s diversity index and with changes in 20% of the bacterial taxa. Multiple oral bacteria tend to be over-represented in the fecal microbiome among PPI users [21].

PPI-induced hypochlorhydria increase the risk of respiratory infection by permitting the transmission of ingested pathogens into the respiratory system. A meta-analysis performed in 2011 found that the risk of community-acquired pneumonia was 34% higher in patients on PPIs, which increased with higher dosing [25].

Long-term use of PPI is associated with small intestinal bacterial overgrowth (SIBO), likely due to hypochlorhydria and loss of gastric defense [26]. According to another meta-analysis, PPI use is associated with an 8-fold relative increased risk of SIBO [27].

PPI could cause profound changes to the colonic microbiota, decrease in the abundance of commensal bacteria, reduce microbial diversity, and increase oral bacteria in stool [21,22,28]. Therefore, PPI-driven dysbiosis increases the risk of enteric infections by *Clostridium difficile*, *Salmonella*, *Campylobacter*, and diarrheagenic *Escherichia coli*.

Retrospective case-control studies show an approximately 3-fold relative risk for *Salmonella* or *Campylobacter* infections after exposure to PPIs [29]. In our study, 10.2% of causative bacteria were *Salmonella* spp. Besides diabetes, autoimmune diseases, cirrhosis, and recent antibiotic use, PPIs have also been associated with *Salmonella* infections [30]. Regarding the possible association between PPI use and bacterial enteric infections, one meta-analysis encompassing over 10,000 patients found a pooled OR of 3.33 [11,15]. A recent meta-analysis showed that use of gastric-acid suppressants may be a risk factor for enteric peritonitis in patients undergoing peritoneal dialysis [4].

PPI use has been linked with increased risk of both incidental and recurrent *Clostridioides difficile* infection (CDi) [31]. According to a systematic review and meta-analysis study, PPI users have a 74% higher risk of developing CDi, as well as a 2.5-fold higher risk of recurrent infections, compared with nonusers [16] Alterations in gut bacteria due to hypochlorhydria may lead to pathogen colonization [21,28]. Seo et al. reports that risk of CDi was significantly greater among groups receiving PPIs and/or H2RAs than among matched controls case (PPIs vs control: HR, 2.65; 95% CI 1.28–5.79; *p* = 0.011; H2RAs vs control: HR 2.43; 95% CI 1.09–5.68; *p* = 0.034) [32]. Wariness of enteric infection is, therefore, warranted for patients on gastric-acid-suppressive drugs and cessation of unnecessary gastric acid suppressive drugs should be considered at the time of CDI diagnosis [31].

PPIs are a risk factor for hepatic encephalopathy and spontaneous bacterial peritonitis in patients with cirrhosis [33]. Studies show a 2-fold relative risk for spontaneous bacterial peritonitis associated with exposure to PPIs [34]. These findings are consistent with the hypothesis that PPIs may increase translocation of gut bacteria and then facilitating the spread of pathogens and bacterial metabolism products. Furthermore, recent study revealed that gut dysbiosis plays an important role in hepatic encephalopathy [35,36]. PPIs use in decompensated cirrhosis is associated with increased risk of mortality and hepatic decompensation [37]. Moreover, PPIs exposure with cDDD > 90 is associated with higher mortality, [aHR = 2.27, (1.10–5.14); P = 0.038, compared to non-users] [38]. In our current study, the adjusted odds ratio (OR) of PPIs use and enteric infection for patient with liver cirrhosis/hepatitis was 3.632 (95% confidence interval [CI], 3.268–4.038).

For the association between PPIs therapy and enteric infection, subgroups with an OR above average were female (OR, 5.565; 95% CI, 5.193–5.964), age between 18~65 year-old (OR 6.252~8.565) and patients with a diagnosis of diabetes mellitus (OR, 5.724, 95% CI, 5.328–6.15). Diabetes mellitus is a risk factor for enteric infection with *Salmonella*, too [30]. Gastric acid suppressive drugs should be used cautionary in diabetic patient. For subgroup of ischemic heart disease, acute myocardial infarction and liver cirrhosis/hepatitis, there is no obvious association between use of H2RAs therapy and enteric infection (OR less than 1). Further prospective investigation is warranted to compare the risk of enteric infection among PPi and H2RAs therapy in such individual.

The strengths of the current study include the large numbers of case patients and controls. The study also had some limitations that need to be considered when interpreting the results. First, it is a retrospective study. PPI or H2RA use was measured using physician prescriptions available in electronic system. Patient compliance with prescribed medication is unknown. Presumes that all medications were taken by the patients as prescribed may overestimate the actual ingested dosage. Patients may not have been identified if their drug was obtained over-the counter. Second, although all results are standardized for age and sex, residual confounding factors are ever present. As acid-suppressive therapy is frequently used in patients with gastrointestinal disorders and in patients prescribed NSAIDs or aspirin, analysis restricted to patients without prior gastrointestinal diseases or prior NSAID exposure was not performed. 

In conclusion, our results suggest that acid-suppressive drugs such as PPIs and H2RAs are associated with an increased risk of enteric infection. More potent acid inhibition is potentially associated with an increased risk. Therefore, acid-suppressive drugs should be prescribed with caution and with full consideration of appropriate dosing and duration.

## Figures and Tables

**Table 1:** Descriptive analysis of population statistics.

Variable	Cases (*N* = 17,186)		Controls ^a^ (*N* = 69,708)		*p* Value
	N/Percent	Mean ± SD	N/Percent	Mean ± SD	
Sex					0.7479 ^a^
Male	8920 (51.9%)	-	36,085 (51.77%)	-	
Female	8266 (48.1%)	-	33,623 (48.23%)	-	
Age, y	-	66.68 ± 17.63	-	66.42 ± 17.58	0.9810 ^a^
18–35	1196 (6.96%)	-	4787 (6.87%)	-	
36–50	1837 (10.69%)	-	7487 (10.74%)	-	
51–65	3936 (22.9%)	-	16,080 (23.07%)	-	
65–80	5736 (33.38%)	-	23,244 (33.34%)	-	
>81	4481 (26.07%)	-	18,110 (25.98%)	-	
PPI Medicine Type	5644 (32.84%)	-	5214 (7.48%)	-	<0.0001
Pantoprazole	1195 (21.17%)	-	1019 (19.54%)	-	
Esomeprazole	3265 (57.85%)	-	2631 (50.46%)	-	
Lansoprazole	1104 (19.56%)	-	1406 (26.97%)	-	
Dexlansoprazole	60 (1.06%)	-	114 (2.19%)	-	
Rabeprazole	20 (0.35%)	-	20 (0.35%)	-	
H2RAs Medicine Type	1709 (9.94%)	-	4836 (6.94%)	-	<0.0001
Famotidine	831 (48.62%)	-	1996 (41.27%)	-	
Cimetidine	878 (51.38%)	-	2840 (58.73%)	-	
PPI or H2RAs Medicine Type	1424 (8.29%)		1907 (2.74%)		<0.0001
Pantoprazole + (Famotidine or Cimetidine)	264 (1.54%)	-	304 (0.44%)	-	
Esomeprazole + (Famotidine or Cimetidine)	790 (4.6%)	-	950 (1.36%)	-	
Lansoprazole + (Famotidine or Cimetidine)	315 (1.83%)	-	540 (0.77%)	-	
Dexlansoprazole + (Famotidine or Cimetidine)	40 (0.23%)	-	83 (0.12%)	-	
Rabeprazole + (Famotidine or Cimetidine)	15 (0.09%)	-	30 (0.04%)	-	
Antibiotic use within past 30 d					<0.0001
Yes	5887 (32.73%)	-	31,460 (43.73%)	-	
No	12,098 (67.27%)	-	40,480 (56.27%)	-	
History of					
Hypertension	9428 (54.86%)	-	41,378 (59.36%)	-	<0.0001
Diabetes mellitus	6353 (36.97%)	-	29,010 (41.62%)	-	<0.0001
Iron deficiency anemia	2067 (12.03%)	-	4916 (7.05%)	-	<0.0001
Ischemic heart disease	1866 (10.86%)	-	5978 (8.58%)	-	<0.0001
Acute myocardial infarction	836 (4.86%)	-	1339 (1.92%)	-	<0.0001
Stroke	2149 (12.5%)	-	5877 (8.43%)	-	<0.0001
Liver cirrhosis/hepatitis	2902 (16.89%)	-	7610 (10.92%)	-	<0.0001
Renal failure	5519 (32.11%)	-	17,447 (25.03%)	-	<0.0001
Gastroesophageal reflux	1839 (10.7%)	-	5596 (8.03%)	-	<0.0001
Gastric ulcer	1670 (9.72%)	-	4151 (5.95%)	-	<0.0001
Duodenal ulcer	1060 (6.17%)	-	2770 (3.97%)	-	<0.0001
Peptic ulcer	3016 (17.55%)	-	8537 (12.25%)	-	<0.0001
Inflammatory bowel disease	200 (1.16%)	-	92 (0.13%)	-	<0.0001
Irritable bowel syndrome	904 (5.26%)	-	2827 (4.06%)	-	<0.0001
Chronic obstruction pulmonary disease	2009 (11.69%)	-	6315 (9.06%)	-	<0.0001
In hospitai all-cause mortality	2494 (14.08%)	-	2694 (3.86%)	-	-

Matched by age, sex, and index year. ^a^ Chi-square test.

**Table 2:** Association between exposure to proton pump inhibitors (PPIs), H2-receptor antagonists (H2RAs), or antibiotic use and intestinal infection.

Exposure	Cases (N = 17,186)	Controls ^a^ (N = 69,708)	Crude OR	Adjusted OR ^b^
	N/Percent	N/Percent	OR (95% CI)	OR (95% CI)
**Among users of PPIs alone** **(*n* = 10,858)**	**5644 (32.84%)**	**5214 (7.48%)**	6.243 (5.98 to 6.518) ***	5.526 (5.274 to 5.791) ***
Drug				
Pantoprazole	1195 (6.95%)	1019 (1.46%)	6.797 (6.232 to 7.413) ***	5.799 (5.297 to 6.349) ***
Esomeprazole	3265 (19%)	2631 (3.77%)	7.133 (6.748 to 7.540) ***	6.319 (5.959 to 6.700) ***
Lansoprazole	1104 (6.42%)	1406 (2.02%)	4.552 (4.195 to 4.940) ***	4.043 (3.709 to 4.407) ***
Dexlansoprazole	60 (0.35%)	114 (0.16%)	2.985 (2.18 to 4.088) ***	2.863 (2.070 to 3.960) ***
Rabeprazole	20 (0.12%)	44 (0.06%)	2.530 (1.489 to 4.298) **	2.112 (1.220 to 3.657) **
Daily done				
cDDD < = 90	2564 (14.92%)	2749 (3.94%)	5.362 (5.061 to 5.680) ***	4.928 (4.639 to 5.236) ***
cDDD 91–180	1647 (9.58%)	1498 (2.15%)	6.388 (5.935 to 6.876) ***	5.637 (5.217 to 6.091) ***
cDDD > 180	1433 (8.34%)	967 (1.39%)	8.545 (7.853 to 9.298) ***	7.153 (6.541 to 7.821) ***
**Among users of ** **H2RAs alone (*n* = 6545)**	**1709 (9.94%)**	**4836 (6.94%)**	1.501 (1.416 to 1.592) ***	1.339 (1.26 to 1.424) ***
Drug				
Famotidine	831 (4.84%)	2840 (4.07%)	1.800 (1.654 to 1.959) ***	1.600 (1.466 to 1.747) **
Cimetidine	878 (5.11%)	1996 (2.86%)	1.303 (1.205 to 1.409) ***	1.164 (1.074 to 1.262) ***
Daily done				
cDDD < = 90	1376 (8.01%)	3997 (5.73%)	1.462 (1.370 to 1.559) ***	1.317 (1.232 to 1.408) ***
cDDD 91–180	242 (1.41%)	664 (0.95%)	1.565 (1.348 to 1.816) ***	1.366 (1.171 to 1.593) ***
cDDD > 180	91 (0.53%)	175 (0.25%)	2.202 (1.704 to 2.847) ***	1.727 (1.323 to 2.255) ***
**Among users of PPIs or** **H2RAs (*n* = 3331)**	**1424 (8.29%)**	**1907 (2.74%)**	3.278 (3.052 to 3.520) ***	2.416 (2.236 to 2.61) ***
Drug				
Pantoprazole + (Famotidine or Cimetidine)	264 (1.54%)	304 (0.44%)	3.761 (3.185 to 4.440) ***	2.712 (2.280 to 3.227) ***
Esomeprazole + (Famotidine or Cimetidine)	790 (4.6%)	950 (1.36%)	3.655 (3.319 to 4.026) ***	2.728 (2.464 to 3.021) ***
Lansoprazole + (Famotidine or Cimetidine)	315 (1.83%)	540 (0.77%)	2.563 (2.227 to 2.950) ***	1.806 (1.557 to 2.096) ***
Dexlansoprazole + (Famotidine or Cimetidine)	40 (0.23%)	83 (0.12%)	2.149 (1.472 to 3.137) ***	1.831 (1.234 to 2.716) *
Rabeprazole + (Famotidine or Cimetidine)	15 (0.09%)	30 (0.04%)	2.231 (1.199 to 4.151) **	1.484 (0.772 to 2.853)
Daily done				
cDDD < = 90	846 (4.92%)	1285 (1.84%)	2.889 (2.643 to 3.157) ***	2.188 (1.991 to 2.405) ***
cDDD 91–180	338 (1.97%)	419 (0.60%)	3.553 (3.073 to 4.108) ***	2.577 (2.213 to 3.000) ***
cDDD > 180	240 (1.4%)	203 (0.29%)	5.242 (4.341 to 6.329) ***	3.561 (2.92 to 4.342) ***
Antibiotic use within past 30 d				
Yes	5653 (32.89%)	30464 (43.7%)	0.627 (0.605 to 0.650) ***	0.623 (0.600 to 0.646) ***
No	11533 (67.11%)	39244 (56.3%)	reference	reference

^a^ Matched by age, sex, and index date. ^b^ Adjusted for by age, sex, and index date, disease history (hypertension, diabetes mellitus iron deficiency anemia, ischemic heart disease, acute myocardial infarction, stroke, liver cirrhosis/hepatitis, renal failure, gastroesophageal reflux, gastric ulcer, duodenal ulcer, peptic ulcer, inflammatory bowel disease, irritable bowel syndrome, chronic obstruction pulmonary disease, gastrointestinal cancer history).* *p* < 0.05, ** *p* < 0.01, *** *p* < 0.001.

**Table 3:** Stratum-specific odds ratios (ORs) for the association between use of proton pump inhibitor therapy and intestinal infection.

Stratum	Cases	Controls ^a^	Crude OR	Adjusted OR ^b^
	Exposed/ Unexposed	Exposed/ Unexposed	OR (95% CI)	AOR (95% CI)
Sex				
Male	3091/5829	2948/33,137	6.118 (5.772 to 6.485) ***	5.476 (5.139 to 5.836) ***
Female	2553/5713	2266/31,357	6.398 (6.001 to 6.822) ***	5.565 (5.193 to 5.964) ***
Age, y				
18–35	161/1035	86/4701	8.601 (6.556 to 11.284) ***	8.565 (6.323 to 11.602) ***
36–50	476/1361	308/7179	8.290 (7.094 to 9.686) ***	7.085 (5.957 to 8.426) ***
51–65	1308/2628	1002/15,078	7.563 (6.894 to 8.296) ***	6.252 (5.650 to 6.919) ***
65–80	2080/3656	2094/21,150	5.778 (5.386 to 6.199) ***	5.264 (4.876 to 5.683) ***
>81	1619/2862	1724/16,386	5.396 (4.987 to 5.838) ***	4.87 (4.474 to 5.301) ***
BMI, kg/m^2^				
<24	1433/2816	1441/12,249	4.424 (4.063 to 4.817) ***	3.923 (3.581 to 4.297) ***
24–30	1019/2078	1200/11,869	4.988 (4.527 to 5.497) ***	4.386 (3.949 to 4.872) ***
>30	218/491	274/3333	5.427 (4.407 to 6.682) ***	4.484 (3.564 to 5.641) ***
History of				
Hypertension	3339/6089	3753/37,625	5.542 (5.249 to 5.851) ***	4.841 (4.566 to 5.133) ***
Diabetes mellitus	2397/3956	2400/26,610	6.695 (6.266 to 7.153) ***	5.724 (5.328 to 6.150) ***
Iron deficiency anemia	817/1250	747/4169	3.737 (3.312 to 4.218) ***	3.646 (3.202 to 4.151) ***
Ischemic heart disease	774/1092	850/5128	4.309 (3.828 to 4.851) ***	3.917 (3.452 to 4.444) ***
Acute myocardial infarction	408/428	292/1047	3.467 (2.857 to 4.206) ***	3.640 (2.970 to 4.461) ***
Stroke	792/1357	767/5110	3.958 (3.518 to 4.452) ***	3.760 (3.315 to 4.265) ***
Liver cirrhosis/hepatitis	1173/1729	1128/6482	3.897 (3.531 to 4.300) ***	3.632 (3.268 to 4.038) ***
Renal failure	2280/3239	1894/15,553	5.916 (5.501 to 6.361) ***	5.517 (5.107 to 5.96) ***
Gastroesophageal reflux	849/990	1424/4172	2.612 (2.335 to 2.922) ***	2.490 (2.216 to 2.797) ***
Gastric ulcer	826/844	1044/3107	3.035 (2.686 to 3.429) ***	2.960 (2.602 to 3.366) ***
Duodenal ulcer	546/514	744/2026	2.979 (2.562 to 3.463) ***	2.909 (2.484 to 3.408) ***
Peptic ulcer	1354/1662	2206/6331	2.356 (2.158 to 2.572) ***	2.208 (2.014 to 2.420) ***
Inflammatory bowel disease	39/161	14/78	1.115 (0.481 to 2.587)	1.387 (0.501 to 3.839)
Irritable bowel syndrome	304/600	482/2345	2.596 (2.180 to 3.092) ***	2.501 (2.072 to 3.019) ***
Chronic obstruction Pulmonary disease	802/1207	862/5453	4.227 (3.765 to 4.747) ***	3.960 (3.499 to 4.482) ***

^a^ Matched by age, sex, and index date. ^b^ Adjusted for by age, sex, and index date, disease history (hypertension, diabetes mellitus, iron deficiency anemia, ischemic heart disease, acute myocardial infarction, stroke, liver cirrhosis/hepatitis, renal failure, gastroesophageal reflux, gastric ulcer, duodenitis ulcer, peptic ulcer, inflammatory bowel disease, irritable bowel syndrome, chronic obstruction pulmonary disease, gastrointestinal cancer history) *** *p* < 0.001.

**Table 4:** Stratum-specific odds ratios (ORs) for the association between use of H2-receptor antagonists therapy and intestinal infection.

Stratum	Cases	Controls ^a^	Crude OR	Adjusted OR ^b^
	Exposed/ Unexposed	Exposed/ Unexposed	OR (95% CI)	AOR (95% CI)
Sex				
Male	842/8078	2332/33,753	1.526 (1.404 to 1.659) ***	1.349 (1.236 to 1.471) ***
Female	867/7399	2504/31,119	1.478 (1.361 to 1.605) ***	1.328 (1.219 to 1.448) ***
Age, y				
18–35	64/1132	84/4703	3.191 (2.287 to 4.452) ***	2.939 (2.062 to 4.191) ***
36–50	163/1674	232/7255	3.074 (2.497 to 3.786) ***	2.563 (2.053 to 3.201) ***
51–65	400/3536	972/15,108	1.772 (1.568 to 2.004) ***	1.557 (1.369 to 1.771) ***
65–80	590/5146	1892/21,352	1.305 (1.183 to 1.44) ***	1.194 (1.078 to 1.322) **
>81	492/3989	1656/16,454	1.238 (1.112 to 1.379) ***	1.117 (0.998 to 1.250)
BMI, kg/m^2^				
<24	556/3693	1471/12,219	1.285 (1.156 to 1.428) ***	1.201 (1.076 to 1.339) ***
24–30	448/2649	1441/11,628	1.367 (1.218 to 1.534) ***	1.289 (1.144 to 1.452) ***
>30	92/617	380/3227	1.188 (0.926 to 1.524)	1.148 (0.886 to 1.489)
History of				
Hypertension	1093/8335	3623/37,755	1.347 (1.252 to 1.448) ***	1.199 (1.112 to 1.292) ***
Diabetes mellitus	684/5669	2192/26,818	1.425 (1.300 to 1.562) ***	1.237 (1.125 to 1.361) ***
Iron deficiency anemia	212/1855	416/4500	1.157 (0.969 to 1.381) ***	1.074 (0.895 to 1.290)
Ischemic heart disease	226/1640	889/5089	0.773 (0.658 to 0.906) **	0.801 (0.680 to 0.943) **
Acute myocardial infarction	81/755	171/1168	0.695 (0.521 to 0.926) *	0.714 (0.532 to 0.960) *
Stroke	289/1860	722/5155	1.093 (0.942 to 1.269)	1.084 (0.929 to 1.265)
Liver cirrhosis/hepatitis	326/2576	924/6686	0.935 (0.816 to 1.072)	0.936 (0.813 to 1.078)
Renal failure	582/4937	1524/15,923	1.194 (1.078 to 1.322) **	1.104 (0.994 to 1.226)
Gastroesophageal reflux	175/1664	764/4832	0.660 (0.553 to 0.787) ***	0.664 (0.554 to 0.795) ***
Gastric ulcer	132/1538	378/3773	0.847 (0.687 to 1.046)	0.819 (0.660 to 1.016)
Duodenal ulcer	66/994	196/2574	0.856 (0.637 to 1.151)	0.792 (0.585 to 1.071)
Peptic ulcer	361/2655	1379/7158	0.722 (0.637 to 0.819) ***	0.746 (0.656 to 0.848) ***
Inflammatory bowel disease	18/182	8/84	0.921 (0.321 to 2.643)	1.018 (0.318 to 3.259)
Irritable bowel syndrome	132/772	454/2373	0.927 (0.747 to 1.149)	0.942 (0.753 to 1.177)
Chronic obstruction pulmonary disease	274/1735	818/5497	1.073 (0.923 to 1.246)	1.040 (0.891 to 1.213)

^a^ Matched by age, sex, and index date. ^b^ Adjusted for by age, sex, and index date, disease history (hypertension, diabetes mellitus, iron deficiency anemia, ischemic heart disease, acute myocardial infarction, stroke, liver cirrhosis/hepatitis, renal failure, gastroesophageal reflux, gastric ulcer, duodenal ulcer, peptic ulcer, inflammatory bowel disease, irritable bowel syndrome, chronic obstruction pulmonary disease, gastrointestinal cancer history) * *p* < 0.05, ** *p* < 0.01, *** *p* < 0.001.

**Table 5:** Isolated microorganisms of the intestinal infection by the stool examination of causative bacteria.

Causative Bacteria	Intestinal Infection (*N* = 8923)	Percentage
*Acineto*	2	0.02%
*Aerobes*	11	0.12%
*Aeromonas*	36	0.40%
*Campy and Campylobacter*	263	2.95%
*Candida*	45	0.50%
*Clostridioides difficile*	3076	34.47%
*Enterococcus*	3995	44.77%
*E.coli*	3	0.03%
*Klebsiella*	10	0.11%
*Plesiomonas shigelloides*	33	0.37%
*Ps. and Pseudomonas* sp.	421	4.72%
*Sal. and Salmonella* sp.	906	10.15%
*Shigella*	14	0.16%
*Staph*	22	0.25%
*Vibrio*	28	0.31%
*Yeast-like*	58	0.65%

## Data Availability

The data presented in this study are available on request from the corresponding author.
